# Superior outcomes with offspring B-leader-matched haploidentical versus older matched sibling donors in AL/MDS patients aged ≥40 years

**DOI:** 10.3389/fimmu.2026.1922699

**Published:** 2026-08-26

**Authors:** Yujun Wei, Jingwen Tang, Ruoling Yang, Yangliu Shao, Songhua Luan, Lu Wang, Lili Wang, Fei Li, Yongli Wu, Kun Qian, Bo Peng, Haoyang Zhang, Dongxue Ge, Yu Fang, Liuqing Huang, Qingyang Liu, Liping Dou, Daihong Liu

**Affiliations:** 1 Department of Hematology, the First Medical Center of Chinese PLA General Hospital, Beijing, China; 2 School of Medicine, Nankai University, Tianjin, China; 3 State Key Laboratory of Experimental Hematology, Senior Department of Hematology, the Fifth Medical Center of PLA General Hospital, Beijing, China

**Keywords:** acute leukemia, haploidentical, hematopoietic stem cell transplantation, HLA-matched sibling, myelodysplastic syndromes

## Abstract

**Introduction:**

With advancing safety profiles in allogeneic hematopoietic stem cell transplantation (allo-HSCT), determining whether HLA-matched siblings remain the optimal donor choice for older adults (≥40 years) and whether haploidentical-HSCT demonstrates superior survival outcomes have become pivotal focuses in transplantation research.

**Methods:**

The goal of this study was to explore the efficacy of haploidentical or matched sibling allo-HSCT in patients over the age of 40 years with acute leukemia (AL) and myelodysplastic syndrome (MDS), with a prespecified primary endpoint of GVHD-free, relapse-free survival (GRFS). In this retrospective study, 195 consecutive AL/MDS patients (≥40 years) who received allo-HSCT between December 2014 and December 2023 were included and analyzed using a 1:2 matched-pair design. All patients received antithymocyte globulin (ATG)-based GVHD prophylaxis. The cohort comprised 130 patients with haploidentical donors (HIDs) and 65 with HLA-matched sibling donors (MSDs).

**Results:**

Patients who received transplants from offspring haploidentical donors with mismatched DRB1 exhibited a significantly lower 5-year cumulative incidence of relapse (15.4%) than those who received MSDs-HSCT (45.3%) or transplants from offspring haploidentical DRB1-matched donors (26.7%; p < 0.001). However, the 5-year nonrelapse mortality (NRM) was highest in the offspring haploidentical B-leader-mismatched group (43.8%) compared with that in the B-leader-matched group (15.4%) and MSDs group (6.3%). Accordingly, in univariate analysis, recipients of offspring haploidentical B-leader-matched donors exhibited superior OS and DFS compared with recipients of older MSDs or offspring B-leader-mismatched donors; multivariate analysis further identified B-leader matching as an independent protective factor against NRM and an independent factor associated with improved GRFS.

**Conclusion:**

In AL/MDS patients aged ≥40 years, offspring haploidentical B-leader-matched donors were associated with favorable survival outcomes compared with older MSDs, primarily driven by reduced NRM; additionally, DRB1 mismatching was independently associated with reduced relapse.

## Introduction

Hematopoietic stem-cell transplantation (HSCT) is a curative option for acute leukemia and myelodysplastic syndrome (MDS) (1). HLA matching is a pivotal determinant of outcomes in HSCT, with an HLA-matched sibling donors (MSDs) representing the optimal donor choice, primarily because of favorable engraftment and lower rates of severe graft-versus-host disease (GVHD) (2). As the number of older adult HSCT recipients has increased (3), a critical challenge has emerged: the readily available matched sibling donors are often also elderly. Extensive research has indicated that donor age is a pivotal determinant of transplant outcomes (2, 4, 5). Data from the European Society for Blood and Marrow Transplantation (EBMT) suggest that donors over 35 years old exhibit poorer nonrelapse mortality (NRM), leukemia-free survival (LFS), and overall survival (OS) in patients over 40 years old (4). Studies have indicated that hematopoietic stem cells from older donors may exhibit potential clonal hematopoiesis (6).

In this context, haploidentical donor (haplo) transplantation has emerged as a universally available alternative (7, 8). Younger donors are believed to provide hematopoietic stem cells with a superior graft-versus-leukemia (GVL) effect (4). A pivotal retrospective analysis demonstrated that in patients aged ≥50 years with acute myeloid leukemia (AML)/MDS, the use of a young haplo donor (≤35 years) resulted in overall survival comparable to that achieved with an older MSDs (≥50 years) (9). Further supporting this finding, a study in older AML patients revealed that while young haplo donors (≤40 years) were associated with different complication profiles (potentially higher NRM), they conferred a significantly lower relapse risk than older MSDs did, leading to comparable long-term survival (8). In another study, Wang et al. (5) performed a multicenter retrospective analysis of 108 CR1 patients who underwent allo-HSCT, revealing that young offspring donors had significantly lower NRM and relapse incidence, resulting in higher OS and LFS compared with older MSDs. These data suggest that the inherent advantages of young haplo donors may offset the risks associated with HLA mismatch.

Moreover, the advantage of younger donors may be closely associated with specific HLA loci (2, 10). A recent large-scale analysis revealed that patients aged 50 years or older who received haploidentical hematopoietic stem cell transplantation (haplo-HSCT) with posttransplantation cyclophosphamide (PTCy) had significantly greater overall survival when the grafts were from young haplo donors (<35 years) with B-leader matching. Additionally, young haplo donors with DRB1 mismatches were associated with a significantly reduced risk of relapse (2). In a cohort receiving anti-thymocyte globulins (ATG)-based haplo-HCT, Jiang et al. (10). further confirmed the association between HLA-B-leader-matched donors and superior outcomes, including reduced NRM and improved OS. These findings suggest that a young, well-matched haplo donor may be preferable to an older, matched sibling donor.

Although previous studies have confirmed the prognostic value of individual HLA-B or DRB1 matching in the PTCy setting, direct comparisons between young haploidentical donors and older matched donors under ATG-based prophylaxis are lacking. It remains unclear whether the advantage of offspring donors in the ATG context is associated with HLA-B-leader matching and DRB1 mismatches. This study explicitly compared young haploidentical donors with older allogeneic hematopoietic stem cell transplant recipients through stratified analysis based on B-leader and DRB1 status, aiming to provide key evidence for donor optimization.

## Materials and methods

### Study design and data collection

In this retrospective, single-center cohort study, we consecutively included 195 patients aged ≥40 years with acute leukemia or MDS who underwent allo-HSCT at our institution between December 2014 and December 2023. The cohort comprised 130 patients who received grafts from haploidentical offspring donors and 65 from matched sibling donors. All clinical data were extracted from our institutional database, which routinely collects follow-up information for all transplant recipients. Patients were tracked from transplantation until death or the end of the follow-up period. The study was conducted in accordance with the Declaration of Helsinki and received approval from the Ethics Committee of the Chinese PLA General Hospital.

### Definitions and evaluation

The prespecified primary endpoint was GVHD-free, relapse-free survival (GRFS). Secondary endpoints included OS, disease-free survival (DFS), cumulative incidence of relapse (CIR), NRM, and acute and chronic graft-versus-host disease (GVHD). Our first aim was to compare the entire haploidentical cohort with the entire MSD cohort. A secondary objective was to compare outcomes among young haploidentical donors with HLA-B-leader matching, those with HLA-B-leader mismatching, and matched sibling donors (MSDs), as well as to evaluate outcomes among young haploidentical donors with HLA-DRB1 matching, those with HLA-DRB1 mismatching, and those with MSDs. Relapse was defined as the reappearance of blasts in the blood in at least 2 peripheral blood samples at least 1 week apart, an increase in blasts to ≥5%, or the development of extramedullary disease after prior achievement of CR (11). GVHD-free, relapse-free survival (GRFS) events were defined as grade 3–4 acute GVHD, chronic GVHD requiring systemic treatment, relapse, or death (12). NRM was defined as death from any cause other than relapse. OS was defined as the time from stem cell infusion to death from any cause (13). DFS was defined as survival without relapse or progression and was calculated until the date of first relapse, death from any cause, or the last follow-up. CR was defined as follows: < 5% bone marrow blasts < 5%, absence of circulating blasts, absence of extramedullary disease, ANC ≥ 1.0 × 10^9^/L (1,000/µL), and platelet count ≥ 100 × 10^9^/L (100,000/µL) (14). Nonremission (NR) was defined as patients who were evaluated for response but did not meet the criteria for complete remission with/without blood count recovery (CR/CRi), and a morphologic leukemia-free state (MLFS) or partial remission (PR) was categorized as having no response prior to the response landmark (14). The grades of acute GVHD were determined with the Mount Sinai Acute GVHD International Consortium (MAGIC) consensus (15), whereas chronic GVHD was diagnosed according to the US National Institute of Health (NIH) criteria (16). Multiparameter flow cytometry (MFC)-measurable residual disease (MRD) test positivity is defined as ≥0.1% of CD45-expressing cells with the target immunophenotype (17). EBV reactivation was defined as a viral load of ≥10,000 copies/mL in a single plasma sample or ≥1000 copies/mL in two consecutive peripheral blood samples. CMV reactivation was defined as a CMV-DNA level ≥10000 copies/mL in a single test or ≥1000 copies/mL in two consecutive tests in plasma (18). Neutrophil engraftment was defined as the first of 3 consecutive days with an absolute neutrop hil count ≥ 0.5 × 10^9^/L. Platelet engraftment was defined as the first of 3 consecutive days with an absolute platelet count ≥ 20 × 10^9^/L without platelet transfusion for 7 days (19). The donor age cutoff of 40 years was selected based on a large EBMT registry study (4) demonstrating that donor age ≥40 years was independently associated with worse outcomes in patients aged ≥40 years undergoing haploidentical transplantation.

### Conditioning regimen

The modified busulfan-cyclophosphamide (mBu/Cy) regimen for haploidentical donor transplantation (HID-HSCT) consisted of cytarabine (4 g/m²/day on days -10 to -9), busulfan (0.8 mg/kg per dose, administered every 6 hours on days -8 to -6; total daily dose 3.2 mg/kg), cyclophosphamide (50 mg/kg/day on days -5 to -4) and ATG (thy moglobulin, rabbit; Sanofi, Paris, France; 10 mg/kg, day - 5 ∼ day - 2). For HLA-matched sibling donor transplantation (MSD-HSCT), the mBu/Cy regimen included cytarabine (4 g/m²/day on day -9), busulfan (3.2 mg/kg/day on days -8 to -6), cyclophosphamide (50 mg/kg/day on days -5 to -4) and ATG (5 mg/kg, day - 5 ∼ day - 2).

The fludarabine-busulfan (FB) regimen for HID-HSCT included fludarabine (30 mg/m²/day on days -7 to -3), busulfan (0.8 mg/kg per dose, administered every 6 hours on days -10 to -8; total daily dose 3.2 mg/kg), cytarabine (1.6 g/m²/day on days -7 to -3), and ATG (thy moglobulin, rabbit; Sanofi, Paris, France; 10 mg/kg, day - 5 ∼ day - 2). For MSD-HSCT, the FB regimen consisted of fludarabine (30 mg/m²/day on days -7 to -3), busulfan (0.8 mg/kg per dose, administered every 6 hours on days -10 to -8; total daily dose 3.2 mg/kg), cytarabine (0.8 g/m²/day on days -7 to -3) and ATG (5 mg/kg, day - 5 ∼ day - 2).

Additionally, selected patients in both groups received the total body irradiation-cyclophosphamide (TBI/Cy) regimen. For patients who underwent haploidentical transplantation, the regimen consisted of TBI (5Gy/day on days -7 to -6, with parotid and lens shielding and partial lung shielding) followed by cyclophosphamide (50 mg/kg/day on days -5 to -4) and ATG (thymoglobulin, rabbit; Sanofi, Paris, France; 10 mg/kg on days -5 to -2). For those receiving HLA-matched sibling donor transplantation, the regimen included the same TBI and cyclophosphamide schedule supplemented with ATG (5 mg/kg on days -5 to -2).

The choice between mBu/Cy and FB regimens was individualized based on patient age, comorbidities, organ function, and disease risk. FB was preferentially used in older patients or those with comorbidities to reduce toxicity, while mBu/Cy was more commonly employed in younger patients or those with high-risk disease (20, 21).

### Supportive care

Cyclosporin A, mycophenolate mofetil, and short-term methotrexate were used for GVHD prophylaxis. Levofloxacin (500 mg) was given orally once daily, starting from the beginning of the conditioning regimen and continuing until day -3. For primary antifungal prophylaxis, either fluconazole or posaconazole was administered at the start of the conditioning regimen. For secondary antifungal prophylaxis, either voriconazole (200 mg, taken orally twice daily) or caspofungin (50 mg per day) was used. All antifungal agents were discontinued approximately six months after transplantation, provided that no fungal infections or pneumonia had occurred. Ganciclovir (250 mg twice daily) was used for cytomegalovirus (CMV) from the start of conditioning to day -3. After graft infusion, preemptive ganciclovir or maribavir was used when CMV-DNA positivity was detected by PCR, with routine monitoring (twice weekly in the first month, weekly thereafter until the third month, and then as needed). Trimethoprim-sulfamethoxazole (SMZ-CO, 960 mg twice daily) was used for *Pneumocystis jirovecii* prophylaxis from the start of conditioning until immunosuppression discontinuation (≥6 months posttransplant). Acyclovir was used for prophylaxis of herpes simplex virus and varicella-zoster virus (VZV) from day +1 until 18 months after transplantation. Supportive care was provided as described previously (22).

### Donor selection and posttransplant follow-up

High-resolution HLA typing was conducted for the HLA-A, -B, -C, -DRB1, and -DQB1 loci. Donor selection prioritized matched sibling donors (MSDs). If an MSD was unavailable, matched unrelated donors were considered. Haploidentical donors (HID) were subsequently considered. Among potential donors, the selection criteria included younger age, better performance status, male sex, and negative donor-specific antibody (DSA) status. All haploidentical donors in our cohort were offspring of the recipients. The term “offspring haploidentical donors” is used throughout the manuscript to refer to all haploidentical donors (n=130), which included 126 donors aged <40 years and 4 donors aged ≥40 years.

All patients were systematically monitored in accordance with a standardized follow-up protocol. Evaluations occurred weekly at the hematology outpatient clinic during the first three months following transplantation, with additional follow-up visits scheduled at 4.5, 6, 9, 12, 18, 24, 36, 48, and 60 months post-transplantation. Patients were advised to seek prompt medical attention for any discomfort or symptoms that may have occurred. MRD status was assessed by multiparameter flow cytometry on bone marrow samples (acquired between 50,000 and 1 million cells) using a leukemia-associated immunophenotype (LAIP) and different-from-normal (DfN) combination approach, with an MRD positivity cutoff of ≥0.1% (23).

### Statistical analysis

We performed 1:2 matched-pair design using the nearest-neighbor algorithm based on patient age (± 2 years). Each patient in the MSDs-HSCT group was matched to two patients who received transplants from young haploidentical donors (offspring). For HLA-matching analysis, “DRB1 mismatch” was defined as the presence of at least a single allele-level mismatch between the donor and recipient at the HLA-DRB1 locus. Similarly, “B-leader match” status was determined on the basis of amino acid sequence identity in the leader peptide region of HLA-B. To investigate the distinct effects of HLA factors within the offspring haploidentical donor cohort, we performed subgroup analyses. Specifically, we assessed the association of B-leader matching with DFS and OS and the association of DRB1 mismatching with relapse risk in patients who received transplants from offspring haploidentical donors. HLA-B leader matching was performed according to the HLA-B leader mismatch calculator (https://www.ebi.ac.uk/ipd/imgt/hla/dpb.html).

Cumulative incidences of relapse, NRM, and GVHD were estimated using the Fine-Gray competing risk model. For CIR, death without relapse was treated as a competing event; for NRM, relapse was considered a competing event. Gray’s test was used for between-group comparisons. Prognostic factors for transplant outcomes were analyzed using univariate and multivariate Cox regression models. OS was estimated using the Kaplan-Meier method and compared using the log-rank test. The proportional hazards (PH) assumption was tested using Schoenfeld residuals for all Cox regression models. For the overall OS comparison between haploidentical and matched sibling donors, where the PH assumption was violated, restricted mean survival time (RMST) analysis was used as an alternative to Cox regression. For subgroup analyses where the PH assumption held (e.g., within the B-leader-matched cohort), Cox regression was retained, with hazard ratios (HRs) and 95% confidence intervals (CIs) reported. Statistical analyses were performed using SPSS 27.0 (SPSS Inc., Chicago, IL, USA) and the R statistical environment v4.4.3. All tests were two-sided, with the type I error rate fixed at 0.05.

## Results

### Patients

In total, 195 consecutive patients aged ≥40 years with acute leukemia or MDS who underwent allo-HSCT at our center between 2014 and 2023 were included in this analysis. No patients were lost to follow-up. The cohort comprised two distinct donor groups: 130 patients (66.7%) who received HIDs-HCT, whereas 65 patients (33.3%) received MSDs-HCT. Among the 130 HID recipients, 98 (75.4%) had B-leader-matched donors and 32 (24.6%) had B-leader-mismatched donors; DRB1 mismatching was present in 115 (88.5%) HID recipients. In the MSDs group, 41 patients (63.1%) achieved CR before transplantation, including 40 patients (61.5%) with a first CR (CR1) and 1 patient (1.5%) with a second CR (CR2). Among HID recipients, 79 patients (60.8%) were in CR1 and 14 (10.8%) were in CR2 at the time of transplantation. Comprehensive baseline characteristics, including patient demographics, disease profiles, and transplant-related parameters, are detailed in **Table 1**.

**Table 1:** Patient and transplant characteristics.

Variable	Matched sibling donor (n=65)	Haploidentical donor (n=130)	*p*
Age of recipient	50 (41-62)	53 (40-73)	0.44
Sex of recipient			0.02
Male, %	35 (53.8%)	92 (70.8%)	
Female, %	30 (46.2%)	38 (29.2%)	
White blood cells≥100×10^9^/L at diagnosis, no (%)	8 (12.3%)	9 (6.9%)	0.21
Diseases			0.39
Acute myeloid leukemia	35 (53.8%)	83 (63.8%)	
Myelodysplastic syndrome-EB	16 (24.6%)	26 (20.0%)	
Acute lymphoblastic leukemia	14 (21.6%)	21 (16.2%)	
Disease Risk Index (DRI)			0.91
Favorable	4 (6.2%)	7 (5.4%)	
Intermediate	30 (46.1%)	58 (44.6%)	
High	31 (47.7%)	65 (50.0%)	
ELN, Risk stratification by genetics in AML	35 (53.8%)	83 (63.8%)	1.00
Favorable	5 (14.3%)	14 (16.9%)	
Intermediate	20 (57.1%)	46 (55.4%)	
Adverse	10 (28.6%)	23 (27.7%)	
IPSS-M of MDS	16 (24.6%)	26 (20.0%)	1.00
Moderate high	1 (6.2%)	1 (3.8%)	
High and very high	15 (93.8%)	25(96.2%)	
Acute lymphoblastic leukemia Cytogenetics	14 (21.6%)	21 (16.2%)	0.70
Normal	4 (28.6%)	3 (14.3%)	
Abnormal	1 (7.1%)	3 (14.3%)	
Ph+	7 (50.0%)	10 (47.6%)	
Others	2 (14.3%)	5 (23.8%)	
Disease status at transplantation, no (%)			0.23
NR	24 (36.9%)	37 (28.5%)	
CR_MRD-positive_	15 (23.1%)	31 (23.8%)	
CR_MRD-negative_	26 (40.0%)	62 (47.7%)	
Conditioning regimen			0.08
mBu/Cy	48 (73.8%)	88 (67.7%)	
FB	14 (21.6%)	41 (31.5%)	
TBI/Cy	3 (4.6%)	1 (0.8%)	
ABO match, no (%)			0.77
Match	30 (46.2%)	59 (45.4%)	
Major mismatch	17 (26.1%)	27 (20.7%)	
Minor mismatch	13 (20.0%)	33(25.4%)	
Bidirectional mismatch	5 (7.7%)	11 (8.5%)	
Donor’s age, %			<0.01
<40y	4(6.2%)	126 (96.9%)	
≥40	61 (93.8%)	4 (3.1%)	
Male donor, %	34 (52.3%)	89 (68.5%)	0.02
HLA characteristics			
DRB1-mismatched, n (%)	0	115 (88.46%)	NA*
B-leader-matched, n (%)	65 (100%)	98 (75.38%)	NA*
Graft			
MNCs, × 10^8^/kg	11.2 (3.0-33.5)	9.4 (2.3-25.6)	0.07
CD34+ cells, × 10^6/^kg	4.1 (1.3-11.4)	4.8 (1.2-16.2)	0.11
Median follow-up time	1771 (795-4027) d	1539 (716-3985) d	

*p-values for HLA variables (DRB1 mismatch and B-leader match) are not applicable (NA) as these variables were part of the donor selection criteria defining the study groups. All other *p*-values were calculated using the chi-square test for categorical variables and the Mann-Whitney U test for continuous variables.

Data are presented as median (range) for continuous variables and as n (%) for categorical variables, unless otherwise indicated. Abbreviations: IPSS-M, Molecular International Prognostic Scoring System; CR, complete remission; NR, nonremission; MNCs, mononuclear cells; MRD, measurable residual disease.

### Engraftment and GVHD

All patients achieved donor cell engraftment. The median time to achieve neutrophil engraftment was 14 days (range 9–29 days) in the HID cohort and 11 days (range 8–29 days) in the MSDs cohort. The 30-day neutrophil engraftment rate was 97.7% (95% CI, 95.0–100%) in the HID cohort and 100% in the MSDs cohort (*p* = 0.55). The 60-day neutrophil engraftment rate was 97.7% (95% CI: 95.0–100%) in the HID cohort and 100% in the MSDs cohort (*p* = 0.55). Among the HID cohort, 3 patients failed to achieve neutrophil engraftment within 30 days, including 3 deaths due to infection. The median time to achieve platelet engraftment was 14 days (range 7–194 days) in the HID cohort and 12 days (range 8–39 days) in the MSDs cohort. The 30-day platelet engraftment rate was 86% (95% CI: 80.0–92.0%) in the HID cohort and 97% (95% CI: 92.0–100%) in the MSDs cohort (*p* = 0.02). The 60-day platelet engraftment rate was 91.1% (95% CI: 86.0–96.0%) in the HID cohort and 100% in the MSDs cohort (*p* = 0.02). Among the HID cohort, 13 patients failed to achieve platelet engraftment within 60 days, including 2 patients with delayed engraftment (at +144 and +194 days posttransplant). The remaining 11 patients died at a median of 35 days (range: 13–185 days), including 7 deaths due to infection, 1 death due to relapse, 2 deaths due to severe aGVHD and 1 death due to hemorrhage.

**Figure 1:**
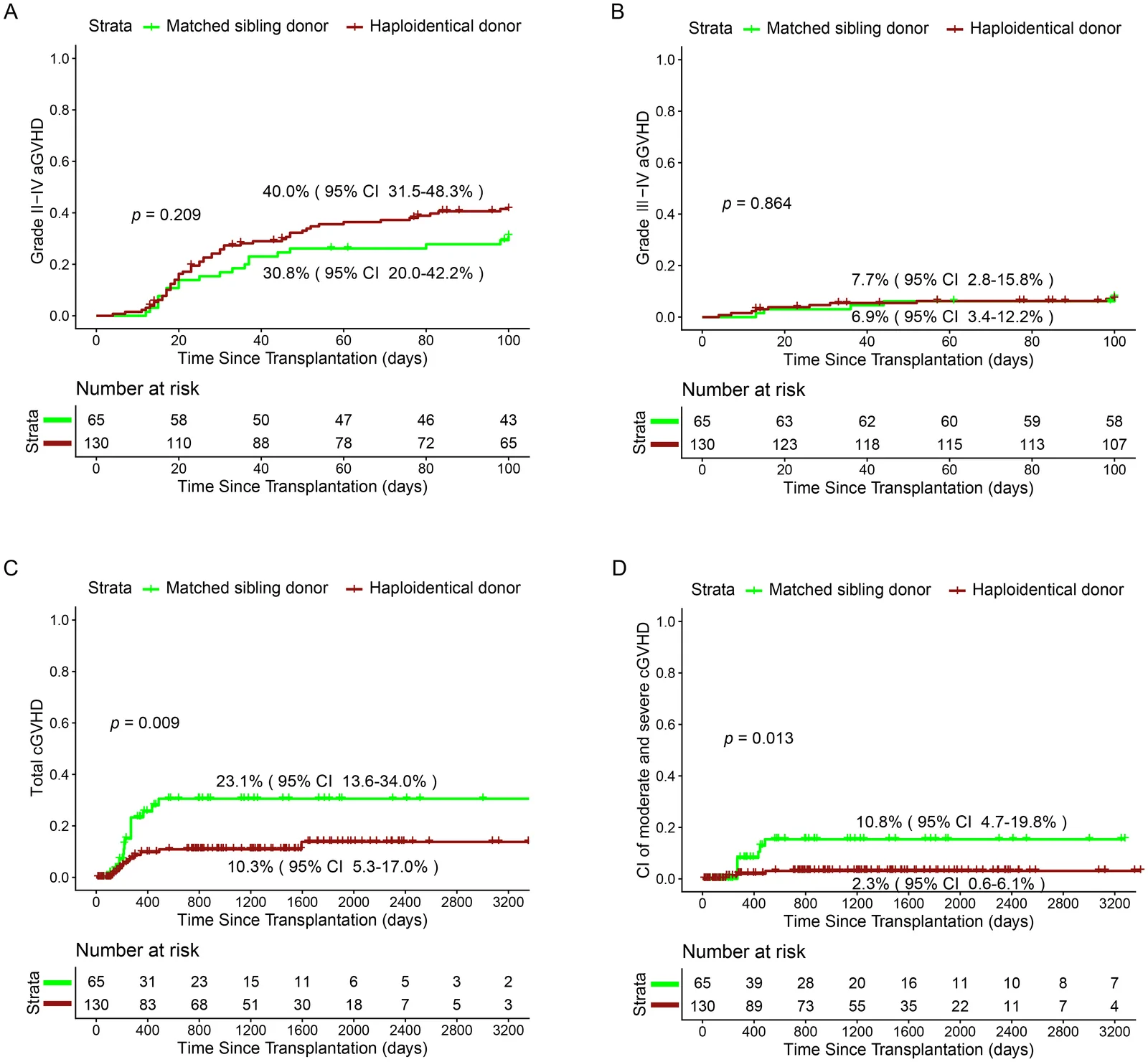
The cumulative incidence of **(A)** Grade II-IV acute graft-versus-host disease (GVDH) between matched sibling donor and haploidentical donor hematopoietic stem cell transplantation (HSCT) (*p* = 0.209); **(B)** Grade III-IV acute GVDH between matched sibling donor and haploidentical donor HSCT (*p* = 0.864); **(C)** total chronic GVHD between matched sibling donor and haploidentical donor HSCT (*p* = 0.009); **(D)** moderate and severe chronic GVHD between matched sibling donor and haploidentical donor HSCT (*p* = 0.013); 95% CI, 95% confidence interval.

The cumulative incidence rates of Grade II–IV aGVHD at 100 days were 40.0% (95% CI: 31.5–48.3%) and 30.8% (95% CI: 20.0–42.2%) after HID and MSDs allo-HSCT, respectively (*p* = 0.209; **Figure 1A**). The cumulative incidences of Grade III–IV aGVHD at 100 days were 7.7% (95% CI: 2.8–15.8%) and 6.9% (95% CI: 3.4–12.2%) after HID and MSDs allo-HSCT, respectively (*p* = 0.864; **Figure 1B**). The cumulative incidences of chronic GVHD at five years were 10.3% (95% CI: 5.3–17.0%) and 23.1% (95% CI: 13.6–34.0%) for HID and MSDs patients, respectively (*p* = 0.009; **Figure 1C**); the cumulative incidence of severe chronic GVHD at five years was 2.3% (95% CI: 0.6–6.1%) for HID patients and 10.8% (95% CI: 4.7–19.8) for MSDs patients (*p* = 0.013; **Figure 1D**). Multivariate analysis revealed that offspring haploidentical B-leader–matched donors (HR = 0.35, 95% CI: 0.14–0.88; *p* = 0.026) was independently associated with a reduced risk of cGVHD (**Supplementary Table 6**), whereas no significant association was observed with aGVHD (**Supplementary Table 5**).

Within 6 months posttransplantation, the cumulative incidence of Epstein–Barr virus (EBV) reactivation was higher in HID recipients than in MSDs recipients (50.8% vs. 29.2%; *p* = 0.004; **Supplementary Figure 1A**), and the cumulative incidence of cytomegalovirus (CMV) reactivation was higher in HID recipients than in MSDs recipients (40.0% vs. 16.9%; *p* = 0.001; **Supplementary Figure 1B**). The incidence of posttranslational lymphoproliferative disorders (PTLDs) within 6 months was 6.2% (n=8) in the HID group and 1.5% (n=1) in the MSDs group (*p* = 0.28).

### Relapse and nonrelapse mortality

**Figure 2:**
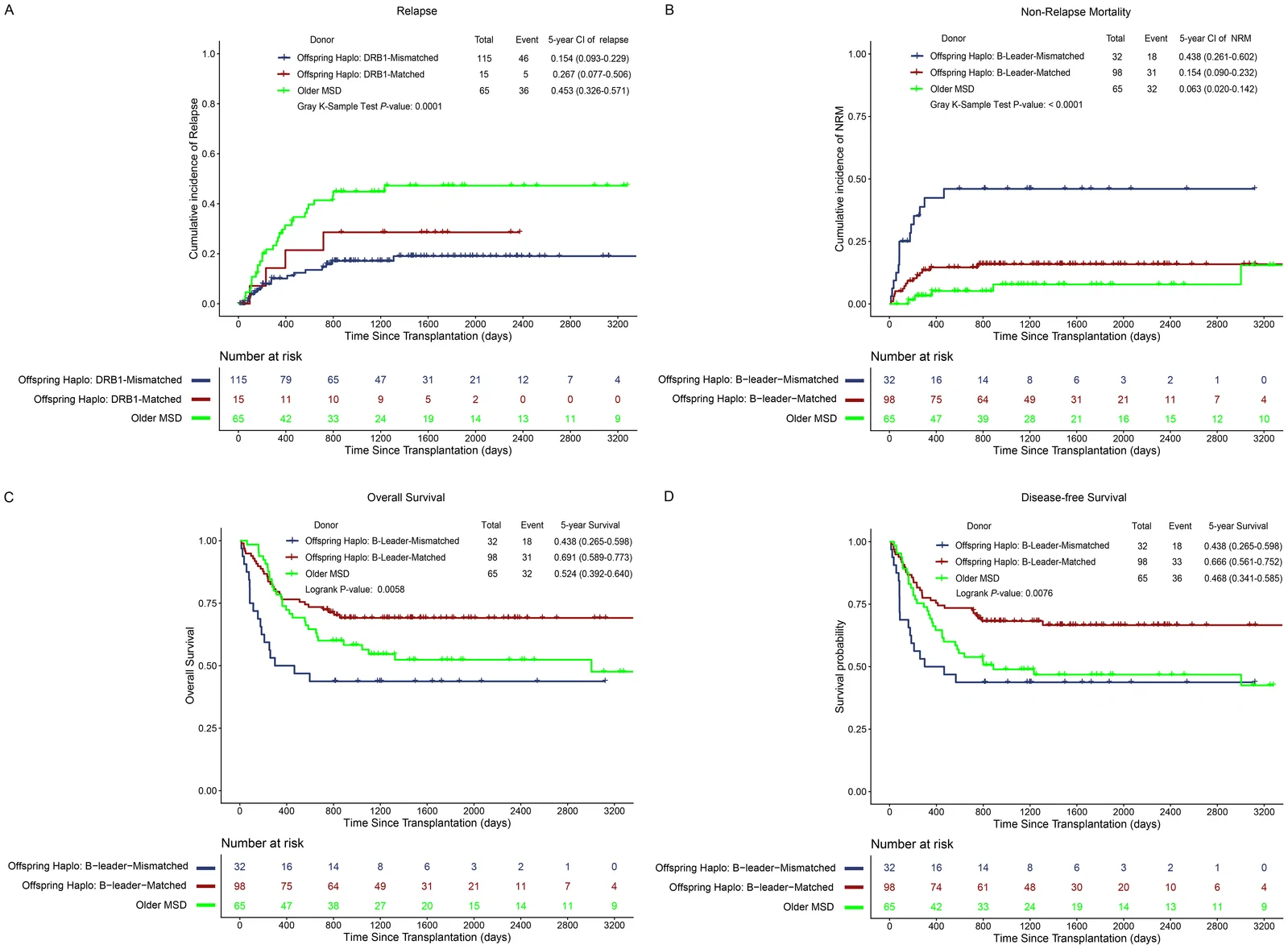
Survival and transplant outcomes by donor type and HLA matching status. **(A)** Cumulative incidence of relapse (CIR). Relapse was compared among three groups: older matched sibling donors (MSDs, green), offspring haploidentical DRB1-matched donors (red), and offspring haploidentical DRB1-mismatched donors (blue). **(B)** Non-relapse mortality (NRM). **(C)** Overall survival (OS). **(D)** Disease-free survival (DFS). For NRM, OS, and DFS, comparisons were made among older MSDs (green), offspring haploidentical B-leader-matched donors (red), and offspring haploidentical B-leader-mismatched donors (blue). *p*-values were calculated using the log-rank test for OS and DFS, and Gray’s test for CIR and NRM. 95% CI, 95% confidence interval.

**Figure 3:**
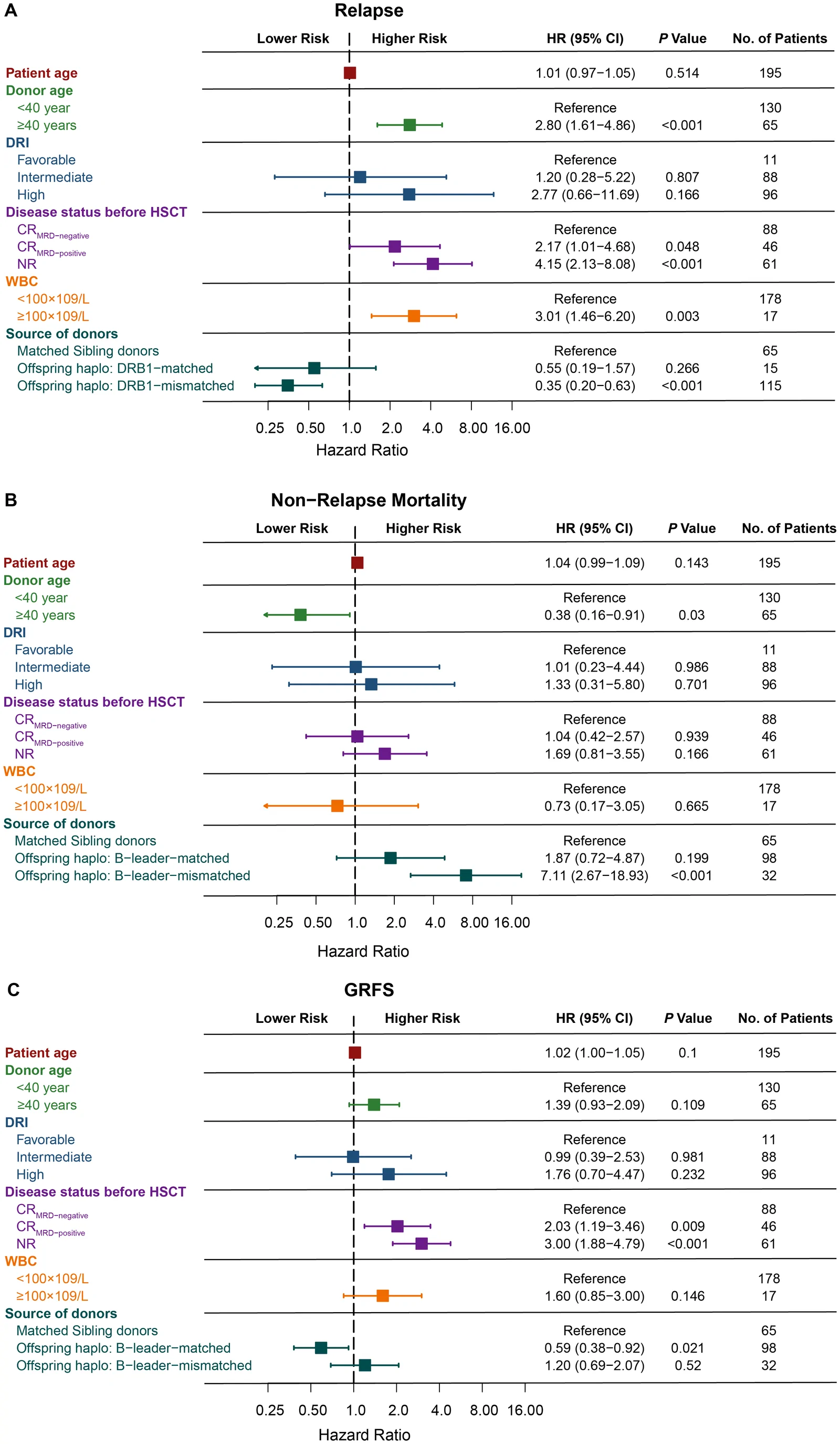
Univariate analysis of factors associated with clinical outcomes. Forest plots showing univariate associations of patient characteristics with **(A)** relapse, **(B)** non-relapse mortality (NRM), and **(C)** GvHD-free, relapse-free survival (GRFS). Hazard ratios (HR) with 95% confidence intervals are presented. Variables with *p* < 0.05 were considered statistically significant. DRI, Disease Risk Index; CR, complete remission; NR, non-remission; MRD, measurable residual disease; WBC, white blood cell; HR, hazard ratio; GRFS, GvHD-free, relapse-free survival.

The 5-year cumulative incidence of relapse was significantly lower in offspring haplo-DRB1-mismatched recipients than in offspring haplo-DRB1- matched recipients and MSDs (15.4% [95% CI: 9.3–22.9] vs. 26.7% [95% CI: 7.7–50.6] vs. 45.3% [95% CI: 32.6–57.1]; *p* = 0.0001; **Figure 2A**; **Supplementary Figure 2A**). Univariate analysis revealed the following factors associated with relapse risk: donor age ≥ 40 years (HR = 2.80; *p* < 0.001), CR_MRD-positive_ (HR = 2.17; *p* = 0.048), NR (HR = 4.15; *p* < 0.001), white blood cells ≥100×10^9^/L (HR = 3.01; *p* = 0.003) and offspring haplo-DRB1-mismatched (HR = 0.35; *p* < 0.001) (**Figure 3A**). Multivariate analysis revealed that NR (HR = 4.17; *p* < 0.001), white blood cell count ≥100×10^9^/L (HR = 3.56; *p* < 0.001) and offspring haplo-DRB1-mismatched (HR = 0.42; *p* = 0.005) were independently associated with increased relapse risk (**Supplementary Table 1**).

The 5-year cumulative incidence rates of NRM were 15.4% (95% CI: 9.0–23.2%), 43.8% (95% CI: 26.1–60.2%) and 6.3% (95% CI: 2.0–14.2) among offspring haplo-B-leader-matched, offspring haplo-B-leader-mismatched and MSDs patients, respectively (*p* < 0.0001; **Figure 2B**; **Supplementary Figure 2B**). In univariate analysis, donor age ≥40 years was associated with a reduced risk of NRM (HR = 0.38; *p* = 0.030), whereas offspring haploidentical B-leader mismatch was associated with an increased risk of NRM (HR = 7.11; *p* < 0.001) (**Figure 3B**). Multivariate analysis incorporating donor age and source revealed that offspring haploidentical B-leader mismatch (HR = 7.11; *p* < 0.001) was an independent risk factor for NRM (**Supplementary Table 2**).

### Overall survival and disease-free survival

After a median follow-up of 1771 days (range, 795–4027), the 5-year probability of OS was 69.1% (95% CI: 58.9–77.3) in offspring haplo-B-leader-matched recipients compared with 43.8% (95% CI: 26.5–59.8) in offspring haplo-B-leader-mismatched recipients and 52.4% (95% CI: 39.2–64.0) in MSDs recipients (*p* = 0.0058; **Figure 2C**; **Supplementary Figure 2C**). The factors associated with OS included NR (HR = 2.79; *p* < 0.001). Multivariate analysis revealed that NR (HR = 2.92; *p* < 0.001) and white blood cell count ≥100×10^9^/L (HR = 2.07; *p* = 0.034) were independently associated with worse OS (**Supplementary Table 3**). Despite similar infection rates at six months (HID: 55.4% vs. MSDs: 53.8%, *p* = 0.53), the proportion of infection-related deaths differed: 38.8% of deaths occurred in the HID group, while 9.1% occurred in the MSDs group (**Table 2**). The causes of death are shown in **Table 3**.

**Table 2:** Outcomes after allogeneic hematopoietic stem cell transplantation.

Variable	Matched sibling donor (n=65)	Haploidentical donor (n=130)	*p*
Engraftment			
Neutrophil engraftment	65 (100%)	127 (97.7%)	0.55
Platelet engraftment	65 (100.0%)	119 (91.5%)	0.02
Infections, no(%)	35 (53.8%)	72 (55.4%)	0.53
Pneumonia, no(%)	20 (30.8%)	36 (27.7%)	
Intestinal infections, no(%)	3 (4.6%)	9 (6.9%)	
Urinary tract infection, no(%)	3 (4.6%)	5 (3.8%)	
Skin and soft tissue infections, no(%)	7 (10.8%)	9 (6.9%)	
Sepsis, no(%)	2 (3.1%)	13 (10.0%)	
Posttransplantation lymphoproliferative disorder, no(%)	1 (1.5%)	8 (6.2%)	0.28
Transplant-associated thrombotic microangiopathy, no(%)	1 (1.5%)	0 (0%)	0.33

**Table 3:** Causes of death.

Cause of death	Matched sibling (n = 33)	Haploidentical donor (n = 49)
Relapse	27 (81.8%)	20 (40.8%)
Infection	3 (9.1%)	19 (38.8%)
Graft-versus-host disease	1 (3.0%)	7 (14.3%)
Hemorrhage	0	2 (4.1%)
Others	2 (6.1%)	1 (2.0%)

The 5-year probability of DFS was 66.6% (95% CI: 56.1–75.2%) in offspring haplo-B-leader-matched recipients, 43.8% (95% CI: 26.5–59.8%) in offspring haplo-B-leader-mismatched recipients and 46.8% (95% CI: 34.1–58.5%) in MSDs patients (*p* = 0.0076) (**Figure 2D**; **Supplementary Figure 2D**). The factors associated with DFS included NR (HR = 2.92; *p* < 0.001), white blood cell count ≥100×10^9^/L (HR = 1.96; *p* = 0.038) and offspring haplo-B-leader-matched (HR = 0.59; *p* = 0.029). Multivariate analysis revealed that NR (HR = 3.24; *p* < 0.001) and white blood cell count ≥100×10^9^/L (HR = 2.18; *p* = 0.018) were independent risk factors associated with poor DFS (**Supplementary Table 4**).

**Figure 4:**
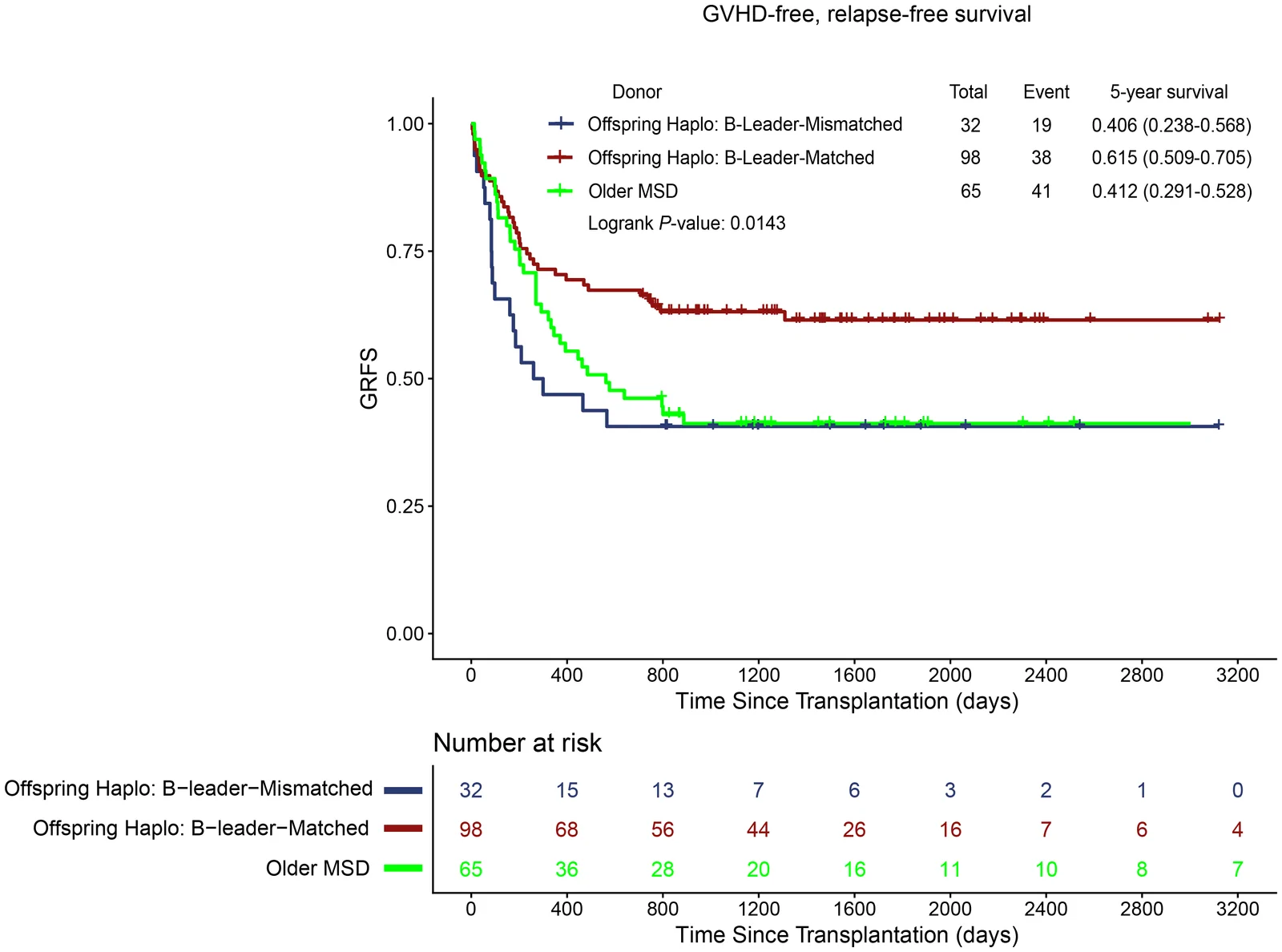
Kaplan–Meier estimates of GRFS for patients receiving transplants from older matched sibling donors (MSDs, green), offspring haploidentical B-leader-matched donors (red), and offspring haploidentical B-leader-mismatched donors (blue). GRFS, GVHD-free, relapse-free survival.

The 5-year probability of GRFS was 61.5% (95% CI: 50.9–70.5%) in the offspring haplo-B-leader-matched group, compared with 40.6% (95% CI: 23.8–56.8%) in the offspring haplo-B-leader-mismatched group and 41.2% (95% CI: 29.1–52.8%) in the MSD group (*p* = 0.0143) (**Figure 4**). In univariate analysis, NR (HR = 3.00; *p* < 0.001) and offspring haplo-B-leader-matched donors (HR = 0.59; *p* = 0.021) were significantly associated with GRFS (**Figure 3C**). Multivariate analysis confirmed NR (HR = 3.68; *p* < 0.001) and offspring haplo-B-leader-matched donors (HR = 0.61; *p* = 0.035) as independent prognostic factors (**Supplementary Table 7**).

## Discussion

This analysis yields two principal findings regarding donor selection for AL/MDS patients aged ≥40 years (24, 25). First, compared with older MSDs, transplantation from offspring haploidentical donors with B-leader matching was associated with superior survival in univariate analysis, with B-leader matching identified as an independent protective factor against NRM and an independent predictor of improved GRFS in multivariate analysis. Second, among patients who underwent haploidentical transplants, a DRB1-mismatched context was associated with a significantly lower relapse risk, underscoring a potent graft-versus-leukemia effect (26). Crucially, this antileukemic benefit must be weighed against the increased NRM observed in B-leader-mismatched pairs (27). Collectively, these findings demonstrate that offspring haploidentical donors with favorable HLA profiles can be preferable alternatives to older MSDs, and they underscore the critical role of integrated HLA evaluation incorporating B-leader matching and DRB1 mismatching in optimizing transplant outcomes (27).

The protective association of B-leader matching with chronic GVHD risk is novel in the context of haploidentical transplantation and suggests a distinct immunomodulatory pathway. Conversely, the association of DRB1 mismatching with potent GVL effects aligns with and extends previous observations on the role of HLA class II disparities in fostering alloreactive immune responses (27, 28). The mechanism by which B-leader matching confers protection may involve modulated allorecognition, whereas DRB1 mismatching likely enhances the breadth of leukemia-associated antigen presentation. This possibility is supported by emerging evidence that specific HLA class II molecules can present cryptic antigens, driving robust alloimmune responses (10, 29). In their retrospective cohort study on unrelated donor hematopoietic-cell transplantation, Petersdorf et al. demonstrated that HLA-B leader dimorphism (M/T) is related to GVHD risk, with leader-matched donors and shared T-leader allotypes being preferred to reduce adverse outcomes, highlighting the critical role of the HLA-B leader in regulating alloreactive NK and T-cell responses through interactions with HLA-E and its receptors (29). Although further stratification by specific B-leader allelic types was not feasible in our cohort due to limited sample size, our findings on B-leader matching are consistent with this mechanistic framework. Consistent with these findings, Wang et al.’s single-center retrospective analysis of haploidentical transplantation with ATG-based conditioning further confirmed that HLA-B leader matching was associated with decreased NRM and improved overall survival, whereas a shared T-leader allotype conferred additional survival benefits, underscoring the translational relevance of HLA-B leader status in donor selection across different transplantation settings (10).

Despite achieving a significantly lower relapse rate, the HID cohort exhibited a marked increase in NRM, with infection-related complications constituting the leading cause of death (38.8% of deaths in HID vs. 9.1% in MSDs). This disparity necessitates mechanistic exploration. First, the more intensive conditioning and immunosuppressive prophylaxis required in haploidentical transplantation, particularly the higher ATG dosage, may lead to prolonged T-cell depletion and delayed immune reconstitution, thereby increasing susceptibility to infections (30). Second, the inherent HLA disparity in haploidentical transplantation not only necessitates intense immunosuppression but also creates a permissive environment for alloreactive responses, contributing to a higher cumulative burden of organ toxicity, infectious complications, and GVHD, all of which are well-established contributors to NRM (31). Our findings provide a clinical correlation to these mechanisms: within the HID cohort, stratification by HLA-B leader matching revealed that offspring recipients of B-leader–mismatched donors faced the highest NRM risk and were independently associated with poorer overall survival. This observation aligns with and extends prior evidence that B-leader matching is linked to improved survival and reduced NRM (2), underscoring its critical role in modulating transplant-related toxicity. Therefore, the elevated NRM in HID transplants appears to stem from a confluence of immune dysregulation and regimen-related toxicity, with specific HLA incompatibilities such as B-leader mismatch pinpointing patients at greatest risk. The higher incidence of CMV/EBV reactivation and PTLD observed in the HID group is directly attributable to the more intensive *in vivo* T-cell depletion strategy employed.

The established prognostic impact of donor age in HSCT provides a critical lens through which to interpret our findings (4, 32). Consistent with prior reports that each rising year of donor age increases mortality risk (33), our multivariate analysis confirmed that older donor age was independently associated with a higher risk of relapse. This phenomenon may be mechanistically linked to the age-related decline in immune competence, as evidenced by a reported fourfold reduction in naïve regulatory T cells (CD45RA+ Tregs) across a donor age range from 20 to 60 years, given that higher levels of these cells are inversely correlated with relapse (34). We observed that the offspring HID group, despite benefiting from offspring donors, received a significantly lower mononuclear cell dose than the MSDs group did. This difference in graft composition may have contributed to higher incidence of infection-related mortality observed in the HID group, thereby offsetting its GVL advantage and partly explaining the comparable OS and DFS between donor types in the overall analysis. Although it is a recognized risk factor for NRM in older patients (32), our analysis did not identify it as an independent predictor, highlighting that offspring haplo B-leader–mismatched factors may exert a more dominant influence on NRM in this patient population. Despite the offsetting effect of infection-related mortality on overall outcomes, the superior GRFS observed in the offspring B-leader-matched group further supports the clinical benefit of this donor selection strategy, as it captures the combined effects of reduced chronic GVHD, preserved GVL activity, and improved survival.

This study has several limitations inherent to its retrospective, single-center design. Heterogeneity in conditioning regimens, and disease status across the groups may have confounded the comparative analyses. Although 1:2 matched-pair design addressed key confounders, unmeasured factors may have influenced the outcomes. The sample size, while sufficient for primary analyses, limits the statistical power for detailed subgroup comparisons, particularly within specific HLA-matching constellations, such as the combination of B-leader matching with DRB1 mismatching. Consequently, our findings regarding the optimal donor–recipient HLA profile require validation in larger, independent cohorts. Furthermore, a major limitation of this study is the near-complete confounding between donor age and donor type. In our cohort, 96.9% of haploidentical donors were aged <40 years, whereas 93.8% of matched sibling donors were aged ≥40 years. This extreme imbalance makes it difficult to fully separate the effect of donor type from that of donor age, and consequently, our findings should be interpreted with caution: the observed “superiority” of haploidentical donors may be at least partly attributable to the younger age of these donors rather than to the donor source per se. Additionally, the superior GVL effect of HID transplantation was counterbalanced by a higher incidence of infection-related mortality, which was closely linked to our immunosuppressive strategy, notably the use of higher ATG doses. These findings underscore the dependence of the net survival benefit on effective management of the infectious risks associated with haploidentical. Future prospective studies should aim to validate the survival advantage of HLA-optimized (B-leader matched) offspring haploidentical donors and to refine conditioning and prophylaxis regimens to mitigate NRM, thereby maximizing the therapeutic index of HID transplantation for older adults with AL/MDS.

## Data Availability

The original contributions presented in the study are included in the article/**Supplementary Material**. Further inquiries can be directed to the corresponding authors.
