# Supplementary material for: Superior outcomes with offspring B-leader-matched haploidentical versus older matched sibling donors in AL/MDS patients aged ≥40 years
Source: Front Immunol. 2026 Aug 26;17:1922699. doi: 10.3389/fimmu.2026.1922699 (PMC13553239; doi:10.3389/fimmu.2026.1922699)
Supplement: Supplementary file 1 [file Supplementaryfile1.docx]

Supplementary Material

# 1 Supplementary Figures and Tables

## 1.1 Supplementary Tables

**Supplementary Table 1 Univariate and multivariate analysis of factors associated with relapse in patients after allo-HSCT.**

| **Variables** | **Univariate analysis** | | | **Multivariate analysis** | | |
| --- | --- | --- | --- | --- | --- | --- |
|  | **HR** | **95%CI** | ***p*** | **HR** | **95%CI** | ***p*** |
| **Patient age** | 1.01 | 0.97-1.05 | 0.514 |  |  |  |
| **Donor age** |  |  |  |  |  |  |
| <40 years | Ref |  |  | Ref |  |  |
| ≥40 years | 2.80 | 1.61-4.86 | ＜0.001 | 1.24 | 0.42-3.68 | 0.703 |
| **Sex** |  |  |  |  |  |  |
| Female | Ref |  |  |  |  |  |
| Male | 1.17 | 0.65-2.09 | 0.605 |  |  |  |
| **Donor Sex** |  |  |  |  |  |  |
| Female | Ref |  |  |  |  |  |
| Male | 1.25 | 0.70-2.20 | 0.453 |  |  |  |
| **Disease Risk Index (DRI)** |  |  |  |  |  |  |
| Favorable | Ref |  |  |  |  |  |
| Intermediate | 1.20 | 0.28-5.22 | 0.807 |  |  |  |
| High | 2.77 | 0.66-11.69 | 0.166 |  |  |  |
| **Disease Type** |  |  |  |  |  |  |
| AML | Ref |  |  |  |  |  |
| MDS-EB | 1.32 | 0.70-2.48 | 0.39 |  |  |  |
| ALL | 0.67 | 0.29-1.52 | 0.334 |  |  |  |
| **Disease status before HSCT** |  |  |  |  |  |  |
| CR_MRD-negative_ | Ref |  |  | Ref |  |  |
| CR_MRD-positive_ | 2.17 | 1.01-4.68 | 0.048 | 2.02 | 0.93-4.36 | 0.075 |
| NR | 4.15 | 2.13-8.08 | ＜0.001 | 4.17 | 2.11-8.26 | ＜0.001 |
| **White blood cells** |  |  |  |  |  |  |
| ＜100×10^9^/L | Ref |  |  | Ref |  |  |
| ≥100×10^9^/L | 3.01 | 1.46-6.20 | 0.003 | 3.56 | 1.68-7.54 | ＜0.001 |
| **MNCs** | 1.03 | 0.98-1.09 | 0.288 |  |  |  |
| **CD34+** | 0.98 | 0.87-1.10 | 0.711 |  |  |  |
| **Source of donors** |  |  |  |  |  |  |
| Matched Sibling donors | Ref |  |  | Ref |  |  |
| Offspring haplo: DRB1-matched | 0.55 | 0.19-1.57 | 0.266 | 0.69 | 0.23-1.98 | 0.485 |
| Offspring haplo: DRB1-mismatched | 0.35 | 0.20-0.63 | ＜0.001 | 0.42 | 0.23-0.77 | 0.005 |

Multivariate Cox regression analysis included variables with *p*<0.10 in univariate analysis.
**Supplementary Table 2 Univariate and multivariate analysis of factors associated with NRM in patients after allo-HSCT.**

| **Variables** | **Univariate analysis** | | | **Multivariate analysis** | | |
| --- | --- | --- | --- | --- | --- | --- |
|  | **HR** | **95%CI** | ***p*** | **HR** | **95%CI** | ***p*** |
| **Patient age** | 1.04 | 0.99-1.09 | 0.143 |  |  |  |
| **Donor age** |  |  |  |  |  |  |
| <40 years | Ref |  |  | Ref |  |  |
| ≥40 years | 0.38 | 0.16-0.91 | 0.030 | 1.02 | 0.15-7.00 | 0.983 |
| **Sex** |  |  |  |  |  |  |
| Female | Ref |  |  |  |  |  |
| Male | 1.09 | 0.54-2.19 | 0.811 |  |  |  |
| **Donor Sex** |  |  |  |  |  |  |
| Female | Ref |  |  |  |  |  |
| Male | 1.21 | 0.60-2.44 | 0.588 |  |  |  |
| **Disease Risk Index (DRI)** |  |  |  |  |  |  |
| Favorable | Ref |  |  |  |  |  |
| Intermediate | 1.01 | 0.23-4.44 | 0.986 |  |  |  |
| High | 1.33 | 0.31-5.80 | 0.701 |  |  |  |
| **Disease Type** |  |  |  |  |  |  |
| AML | Ref |  |  |  |  |  |
| MDS-EB | 1.36 | 0.82-2.26 | 0.230 |  |  |  |
| ALL | 0.77 | 0.41-1.45 | 0.420 |  |  |  |
| **Disease status before HSCT** |  |  |  |  |  |  |
| CR_MRD-negative_ | Ref |  |  |  |  |  |
| CR_MRD-positive_ | 1.04 | 0.42-2.57 | 0.939 |  |  |  |
| NR | 1.69 | 0.81-3.55 | 0.166 |  |  |  |
| **White blood cells** |  |  |  |  |  |  |
| ＜100×10^9^/L | Ref |  |  |  |  |  |
| ≥100×10^9^/L | 0.73 | 0.17-3.05 | 0.665 |  |  |  |
| **MNCs** | 0.98 | 0.90-1.06 | 0.563 |  |  |  |
| **CD34+** | 1.06 | 0.94-1.19 | 0.316 |  |  |  |
| **Source of donors** |  |  |  |  |  |  |
| Matched Sibling donors | Ref |  |  | Ref |  |  |
| Offspring haplo: B-leader–matched | 1.87 | 0.72-4.87 | 0.199 | 1.87 | 0.72-4.87 | 0.199 |
| Offspring haplo: B-leader–mismatched | 7.11 | 2.67-18.93 | ＜0.001 | 7.11 | 2.67-18.93 | ＜0.001 |

Multivariate Cox regression analysis included variables with *p*<0.10 in univariate analysis.

**Supplementary Table 3 Univariate and multivariate analysis of factors associated with OS in patients after allo-HSCT.**

| **Variables** | **Univariate analysis** | | | **Multivariate analysis** | | |
| --- | --- | --- | --- | --- | --- | --- |
|  | **HR** | **95%CI** | ***p*** | **HR** | **95%CI** | ***p*** |
| **Patient age** | 1.03 | 0.99-1.06 | 0.126 |  |  |  |
| **Donor age** |  |  |  |  |  |  |
| <40 years | Ref |  |  |  |  |  |
| ≥40 years | 1.23 | 0.79-1.92 | 0.366 |  |  |  |
| **Sex** |  |  |  |  |  |  |
| Female | Ref |  |  |  |  |  |
| Male | 1.21 | 0.76-1.94 | 0.416 |  |  |  |
| **Donor Sex** |  |  |  |  |  |  |
| Female | Ref |  |  |  |  |  |
| Male | 1.22 | 0.77-1.92 | 0.402 |  |  |  |
| **Disease Risk Index (DRI)** |  |  |  |  |  |  |
| Favorable | Ref |  |  |  |  |  |
| Intermediate | 1.03 | 0.36-2.93 | 0.959 |  |  |  |
| High | 1.84 | 0.66-5.15 | 0.247 |  |  |  |
| **Disease Type** |  |  |  |  |  |  |
| AML | Ref |  |  |  |  |  |
| MDS-EB | 1.36 | 0.82-2.26 | 0.230 |  |  |  |
| ALL | 0.77 | 0.41-1.45 | 0.420 |  |  |  |
| **Disease status before HSCT** |  |  |  |  |  |  |
| CR_MRD-negative_ | Ref |  |  | Ref |  |  |
| CR_MRD-positive_ | 1.60 | 0.88-2.91 | 0.121 | 1.60 | 0.88-2.91 | 0.123 |
| NR | 2.79 | 1.68-4.64 | ＜0.001 | 2.92 | 1.75-4.88 | ＜0.001 |
| **White blood cells** |  |  |  |  |  |  |
| ＜100×10^9^/L | Ref |  |  | Ref |  |  |
| ≥100×10^9^/L | 1.78 | 0.92-3.46 | 0.089 | 2.07 | 1.06-4.04 | 0.034 |
| **MNCs** | 1.01 | 0.96-1.06 | 0.683 |  |  |  |
| **CD34+** | 1.02 | 0.94-1.11 | 0.614 |  |  |  |
| **Source of donors*** |  |  |  |  |  |  |
| Matched Sibling donors | Ref |  |  |  |  |  |
| Offspring haplo: B-leader–matched | +175.7** | -49.00-400.4 | 0.125 |  |  |  |
| Offspring haplo: B-leader–mismatched | -282.79 | -617.80-52.21 | 0.098 |  |  |  |

*For variables other than donor type, effect estimates are presented as hazard ratios (HR) from Cox proportional hazards regression. For donor type, estimates are presented as RMST differences (in days) due to violation of the proportional hazards assumption.

**Multivariate Cox regression analysis included variables with *p*<0.10 in univariate analysis. Donor type was not included in the multivariate Cox model due to PH violation; its independent effect was assessed using covariate-adjusted RMST analysis and is reported in the text (Results section)

**Supplementary Table 4 Univariate and multivariate analysis of factors associated with DFS in patients after allo-HSCT.**

| **Variables** | **Univariate analysis** | | | **Multivariate analysis** | | |
| --- | --- | --- | --- | --- | --- | --- |
|  | **HR** | **95%CI** | ***p*** | **HR** | **95%CI** | ***p*** |
| **Patient age** | 1.02 | 0.99-1.05 | 0.161 |  |  |  |
| **Donor age** |  |  |  |  |  |  |
| <40 years | Ref |  |  |  |  |  |
| ≥40 years | 1.43 | 0.93-2.19 | 0.103 |  |  |  |
| **Sex** |  |  |  |  |  |  |
| Female | Ref |  |  |  |  |  |
| Male | 1.12 | 0.72-1.76 | 0.607 |  |  |  |
| **Donor Sex** |  |  |  |  |  |  |
| Female | Ref |  |  |  |  |  |
| Male | 1.24 | 0.79-1.92 | 0.350 |  |  |  |
| **Disease Risk Index (DRI)** |  |  |  |  |  |  |
| Favorable | Ref |  |  |  |  |  |
| Intermediate | 1.09 | 0.38-3.08 | 0.875 |  |  |  |
| High | 1.99 | 0.72-5.55 | 0.188 |  |  |  |
| **Disease Type** |  |  |  |  |  |  |
| AML | Ref |  |  |  |  |  |
| MDS-EB | 1.38 | 0.85-2.25 | 0.196 |  |  |  |
| ALL | 0.77 | 0.42-1.42 | 0.400 |  |  |  |
| **Disease status before HSCT** |  |  |  |  |  |  |
| CR_MRD-negative_ | Ref |  |  | Ref |  |  |
| CR_MRD-positive_ | 1.58 | 0.89-2.82 | 0.122 | 1.74 | 0.97-3.13 | 0.063 |
| NR | 2.92 | 1.79-4.77 | ＜0.001 | 3.24 | 1.96-5.35 | ＜0.001 |
| **White blood cells** |  |  |  |  |  |  |
| ＜100×10^9^/L | Ref |  |  | Ref |  |  |
| ≥100×10^9^/L | 1.96 | 1.04-3.70 | 0.038 | 2.18 | 1.15-4.16 | 0.018 |
| **MNCs** | 1.01 | 0.97-1.06 | 0.636 |  |  |  |
| **CD34+** | 1.01 | 0.93-1.10 | 0.774 |  |  |  |
| **Source of donors** |  |  |  |  |  |  |
| Matched Sibling donors | Ref |  |  | Ref |  |  |
| Offspring haplo: B-leader–matched | 0.59 | 0.37-0.95 | 0.029 | 0.67 | 0.41-1.08 | 0.097 |
| Offspring haplo: B-leader–mismatched | 1.37 | 0.77-2.42 | 0.284 | 1.75 | 0.98-3.13 | 0.060 |

Multivariate Cox regression analysis included variables with *p*<0.10 in univariate analysis.

**Supplementary Table 5 Univariate and multivariate analysis of factors associated with Ⅱ-Ⅳ acute GVHD in patients after allo-HSCT.**

| **Variables** | **Univariate analysis** | | | **Multivariate analysis** | | |
| --- | --- | --- | --- | --- | --- | --- |
|  | **HR** | **95%CI** | ***p*** | **HR** | **95%CI** | ***p*** |
| **Patient age** | 1.00 | 0.97-1.03 | 0.964 |  |  |  |
| **Donor age** |  |  |  |  |  |  |
| <40 years | Ref |  |  |  |  |  |
| ≥40 years | 0.69 | 0.411-1.16 | 0.157 |  |  |  |
| **Sex** |  |  |  |  |  |  |
| Female | Ref |  |  |  |  |  |
| Male | 1.02 | 0.63-1.66 | 0.926 |  |  |  |
| **Donor Sex** |  |  |  |  |  |  |
| Female | Ref |  |  |  |  |  |
| Male | 1.20 | 0.74-1.96 | 0.466 |  |  |  |
| **Disease Risk Index (DRI)** |  |  |  |  |  |  |
| Favorable | Ref |  |  |  |  |  |
| Intermediate | 1.61 | 0.50-5.25 | 0.427 |  |  |  |
| High | 1.43 | 0.44-4.66 | 0.551 |  |  |  |
| **Disease Type** |  |  |  |  |  |  |
| AML | Ref |  |  |  |  |  |
| MDS-EB | 0.97 | 0.55-1.73 | 0.929 |  |  |  |
| ALL | 0.91 | 0.49-1.69 | 0.759 |  |  |  |
| **Disease status before HSCT** |  |  |  |  |  |  |
| CR_MRD-negative_ | Ref |  |  |  |  |  |
| CR_MRD-positive_ | 1.28 | 0.70-2.34 | 0.419 |  |  |  |
| NR | 1.49 | 0.88-2.53 | 0.138 |  |  |  |
| **White blood cells** |  |  |  |  |  |  |
| ＜100×10^9^/L | Ref |  |  |  |  |  |
| ≥100×10^9^/L | 0.99 | 0.43-2.27 | 0.973 |  |  |  |
| **MNCs** | 0.99 | 0.95-1.04 | 0.815 |  |  |  |
| **CD34+** | 0.97 | 0.88-1.07 | 0.487 |  |  |  |
| **Source of donors** |  |  |  |  |  |  |
| Matched Sibling donors | Ref |  |  |  |  |  |
| Offspring haplo: B-leader–matched | 1.38 | 0.80-2.37 | 0.243 |  |  |  |
| Offspring haplo: B-leader–mismatched | 1.62 | 0.82-3.22 | 0.165 |  |  |  |

Multivariate Cox regression analysis included variables with *p*<0.10 in univariate analysis.

**Supplementary Table 6 Univariate and multivariate analysis of factors associated with chronic GVHD in patients after allo-HSCT.**

| **Variables** | **Univariate analysis** | | | **Multivariate analysis** | | |
| --- | --- | --- | --- | --- | --- | --- |
|  | **HR** | **95%CI** | ***p*** | **HR** | **95%CI** | ***p*** |
| **Patient age** | 0.98 | 0.93-1.04 | 0.465 |  |  |  |
| **Donor age** |  |  |  |  |  |  |
| <40 years | Ref |  |  |  |  |  |
| ≥40 years | 1.74 | 0.81-3.71 | 0.155 |  |  |  |
| **Sex** |  |  |  |  |  |  |
| Female | Ref |  |  |  |  |  |
| Male | 0.93 | 0.43-2.03 | 0.857 |  |  |  |
| **Donor Sex** |  |  |  |  |  |  |
| Female | Ref |  |  |  |  |  |
| Male | 0.71 | 0.33-1.51 | 0.374 |  |  |  |
| **Disease Risk Index (DRI)** |  |  |  |  |  |  |
| Favorable | Ref |  |  |  |  |  |
| Intermediate | 0.86 | 0.20-3.80 | 0.844 |  |  |  |
| High | 0.75 | 0.17-3.38 | 0.703 |  |  |  |
| **Disease Type** |  |  |  |  |  |  |
| AML | Ref |  |  | Ref |  |  |
| MDS-EB | 2.40 | 1.01-5.71 | 0.047 | 1.83 | 0.52-6.44 | 0.350 |
| ALL | 1.56 | 0.59-4.16 | 0.373 | 2.17 | 0.72-6.53 | 0.170 |
| **Disease status before HSCT** |  |  |  |  |  |  |
| CR_MRD-negative_ | Ref |  |  |  |  |  |
| CR_MRD-positive_ | 1.86 | 0.72-4.82 | 0.202 |  |  |  |
| NR | 2.37 | 0.96-5.85 | 0.061 |  |  |  |
| **White blood cells** |  |  |  |  |  |  |
| ＜100×10^9^/L | Ref |  |  |  |  |  |
| ≥100×10^9^/L | 0.48 | 0.07-3.52 | 0.468 |  |  |  |
| **MNCs** | 0.92 | 0.83-1.02 | 0.096 | 0.90 | 0.81-1.01 | 0.080 |
| **CD34+** | 0.81 | 0.66-1.00 | 0.046 | 0.86 | 0.68-1.08 | 0.194 |
| **Source of donors** |  |  |  |  |  |  |
| Matched Sibling donors | Ref |  |  | Ref |  |  |
| Offspring haplo: B-leader–matched | 0.68 | 0.23-2.06 | 0.500 | 0.35 | 0.14-0.88 | 0.026 |
| Offspring haplo: B-leader–mismatched | 0.29 | 0.12-0.69 | 0.005 | 0.68 | 0.21-2.18 | 0.512 |

Multivariate Cox regression analysis included variables with *p*<0.10 in univariate analysis.

**Supplementary Table 7 Univariate and multivariate analysis of factors associated with chronic GRFS in patients after allo-HSCT.**

| **Variables** | **Univariate analysis** | | | **Multivariate analysis** | | |
| --- | --- | --- | --- | --- | --- | --- |
|  | **HR** | **95%CI** | ***p*** | **HR** | **95%CI** | ***p*** |
| **Patient age** | 1.02 | 1.00-1.05 | 0.100 | 1.02 | 0.99-1.05 | 0.158 |
| **Donor age** |  |  |  |  |  |  |
| <40 years | Ref |  |  |  |  |  |
| ≥40 years | 1.39 | 0.93-2.09 | 0.109 |  |  |  |
| **Sex** |  |  |  |  |  |  |
| Female | Ref |  |  |  |  |  |
| Male | 1.02 | 0.68-1.55 | 0.911 |  |  |  |
| **Donor Sex** |  |  |  |  |  |  |
| Female | Ref |  |  |  |  |  |
| Male | 1.06 | 0.70-1.60 | 0.776 |  |  |  |
| **Disease Risk Index (DRI)** |  |  |  |  |  |  |
| Favorable | Ref |  |  |  |  |  |
| Intermediate | 0.99 | 0.39-2.53 | 0.981 |  |  |  |
| High | 1.76 | 0.70-4.47 | 0.232 |  |  |  |
| **Disease Type** |  |  |  |  |  |  |
| AML | Ref |  |  | Ref |  |  |
| MDS-EB | 1.51 | 0.95-2.39 | 0.081 | 0.65 | 0.37-1.16 | 0.149 |
| ALL | 0.78 | 0.44-1.37 | 0.386 | 0.92 | 0.50-1.68 | 0.781 |
| **Disease status before HSCT** |  |  |  |  |  |  |
| CR_MRD-negative_ | Ref |  |  | Ref |  |  |
| CR_MRD-positive_ | 2.03 | 1.19-3.46 | 0.009 | 2.16 | 1.24-3.78 | 0.007 |
| NR | 3.00 | 1.88-4.79 | <0.001 | 3.68 | 2.03-6.68 | <0.001 |
| **White blood cells** |  |  |  |  |  |  |
| ＜100×10^9^/L | Ref |  |  |  |  |  |
| ≥100×10^9^/L | 1.60 | 0.85-3.00 | 0.146 |  |  |  |
| **MNCs** | 1.00 | 0.96-1.05 | 0.943 |  |  |  |
| **CD34+** | 0.98 | 0.90-1.07 | 0.660 |  |  |  |
| **Source of donors** |  |  |  |  |  |  |
| Matched Sibling donors | Ref |  |  | Ref |  |  |
| Offspring haplo: B-leader–matched | 0.59 | 0.38-0.92 | 0.021 | 0.61 | 0.39-0.97 | 0.035 |
| Offspring haplo: B-leader–mismatched | 1.20 | 0.69-2.07 | 0.520 | 1.40 | 0.80-2.46 | 0.235 |

Multivariate Cox regression analysis included variables with *p*<0.10 in univariate analysis.

## 1.2 Supplementary Figures

**Supplementary Figure 1.** The 6 months cumulative incidence of (A) Epstein–Barr virus (EBV) between matched sibling donor and offspring donor HSCT (*p*=0.004); (B) Cytomegalovirus (CMV) between matched sibling donor and offspring donor hematopoietic stem cell transplantation (HSCT) (*p*=0.001).

**Supplementary Figure 2.** Five-year post-transplantation outcomes by donor type in acute leukemia/MDS patients. (A) Cumulative incidence of relapse: Matched sibling donor vs. haploidentical donor HSCT (*p* < 0.001). (B) Cumulative incidence of nonrelapse mortality (NRM): Matched sibling donor vs. haploidentical donor HSCT (*p*=0.009). (C) Probability of overall survival (OS): Matched sibling donor vs. haploidentical donor HSCT (*p* = 0.510). (D) Probability of disease-free survival (DFS): Matched sibling donor vs. haploidentical donor HSCT (*p* = 0.160); 95% CI, 95% confidence interval.

**Supplementary Figure 1**

**Supplementary Figure 2**
